# Supplementary material for: Ultra-deep sequencing reveals dramatic alteration of organellar genomes in Physcomitrella patens due to biased asymmetric recombination
Source: Commun Biol. 2021 May 27;4:633. doi: 10.1038/s42003-021-02141-x (PMC8159992; doi:10.1038/s42003-021-02141-x)
Supplement: Supplementary file 2 — Supplementary Information [file 42003_2021_2141_MOESM2_ESM.pdf]

Supplementary Information

Ultra-deep sequencing reveals dramatic alteration of organellar genomes in *Physcomitrella patens* due to biased asymmetric recombination

Masaki Odahara, Kensuke Nakamura, Yasuhiko Sekine, and Taku Oshima

Supplementary Figure 1-15
Supplementary Table 1-11
Supplementary References
Supplementary Note (README)

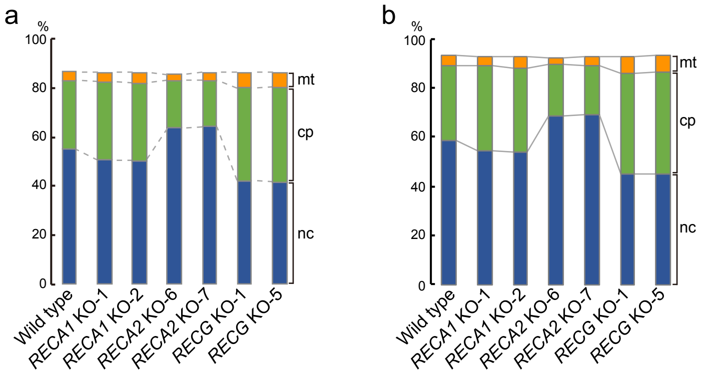

Supplementary Figure 1. Mapping of reads to nuclear, chloroplast, and mitochondrial DNA.

a. Mapping rate of Illumina reads (150 bp) with 2 bp mismatch permitted (h2r3).

b. Mapping rate of truncated reads (100 bp) with 2 bp mismatch permitted (chp100_h2r3).

Large portion of the unmapped reads in (A) and (B) should be reads including sequencing errors.

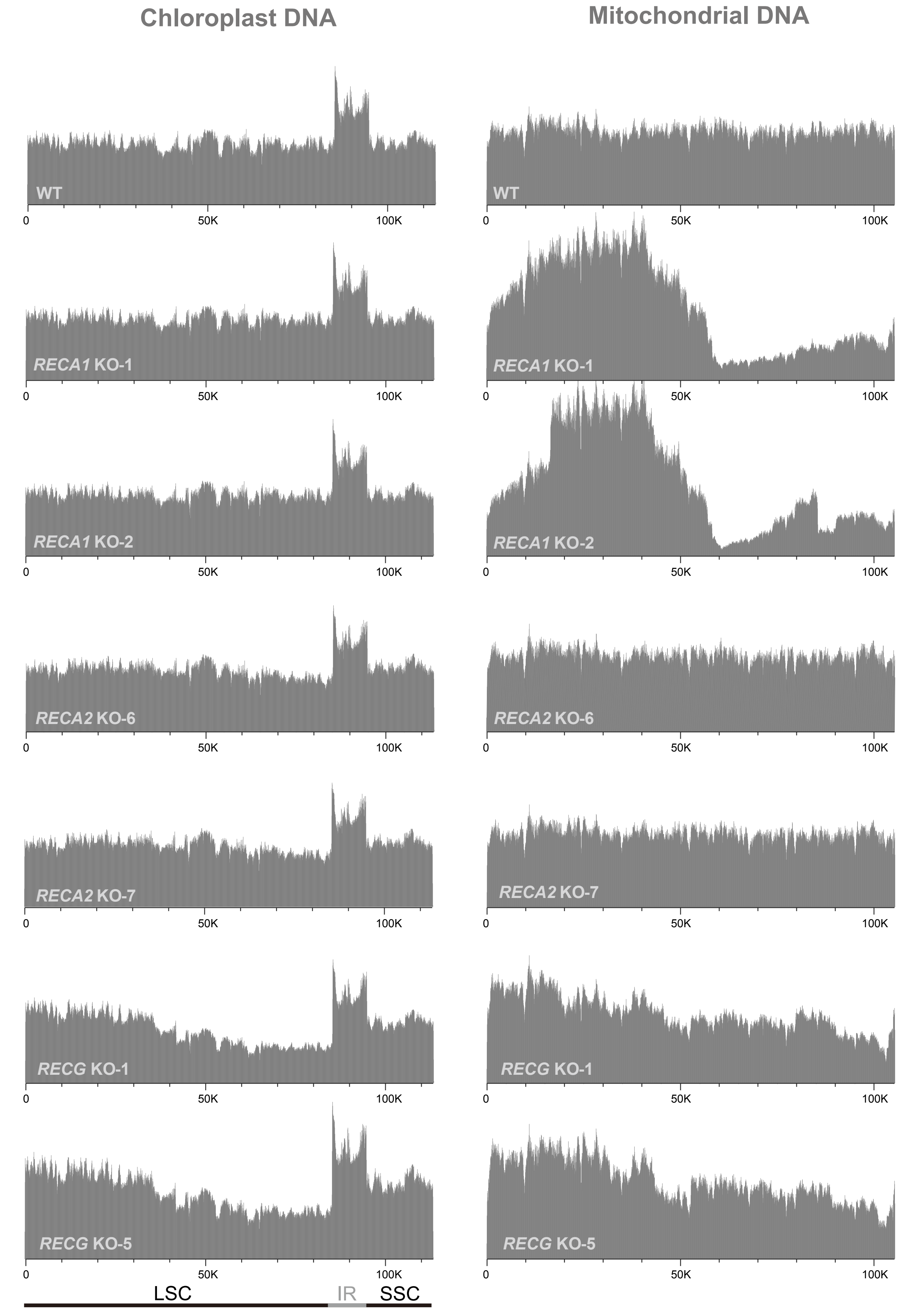

Supplementary Figure 2. Mapping reads to *P. patens* organelle genomes.
Reads are mapped to chloroplast and mitochondrial DNA by mpsmap. Large single copy, small single copy, and inverted repeat of chloroplast DNA are denoted by LSC, SSC, and IR, respectively. One copy of IR is removed from the chloroplast DNA reference sequence.

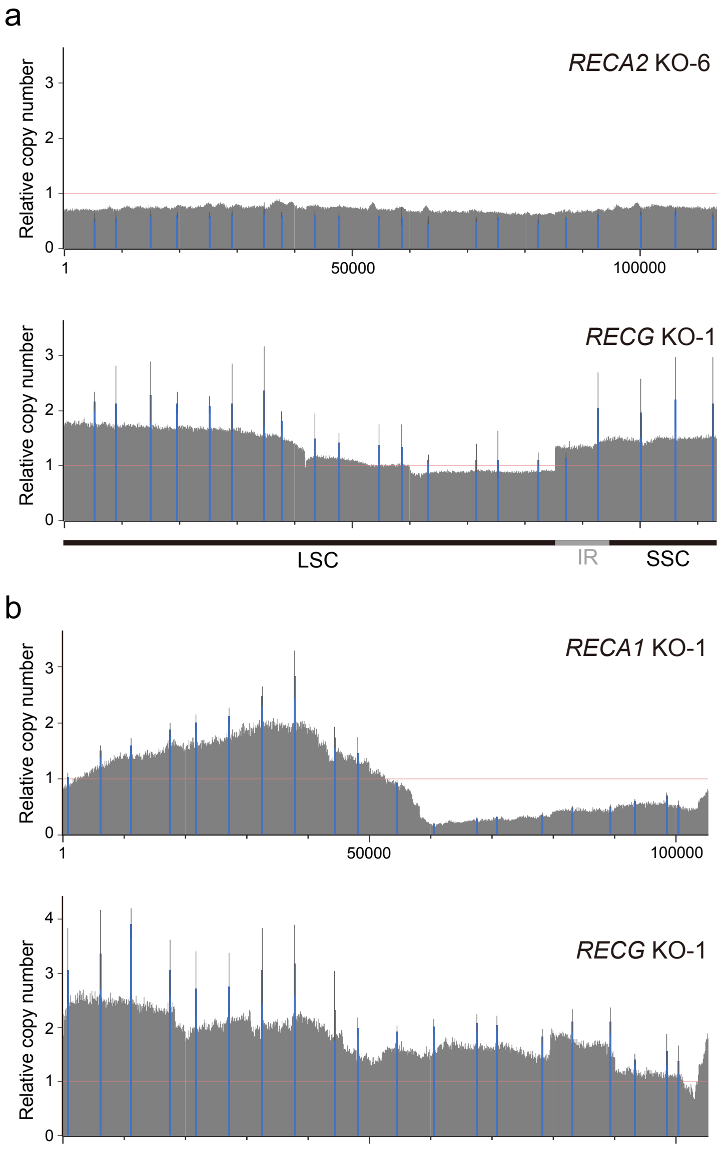

Supplementary Figure 3. Quantitative PCR evaluation of read depth.
Normalized read depth of mutant's organelle DNA shown in Figure 2 were compared with results from quantitative PCR analyses of each organelle loci normolized with that of nuclear DNA. Copy number at each locus of cpDNA (**a**) and mtDNA (**b**) relative to wild type are shown by blue bars with black error bars (n=3).

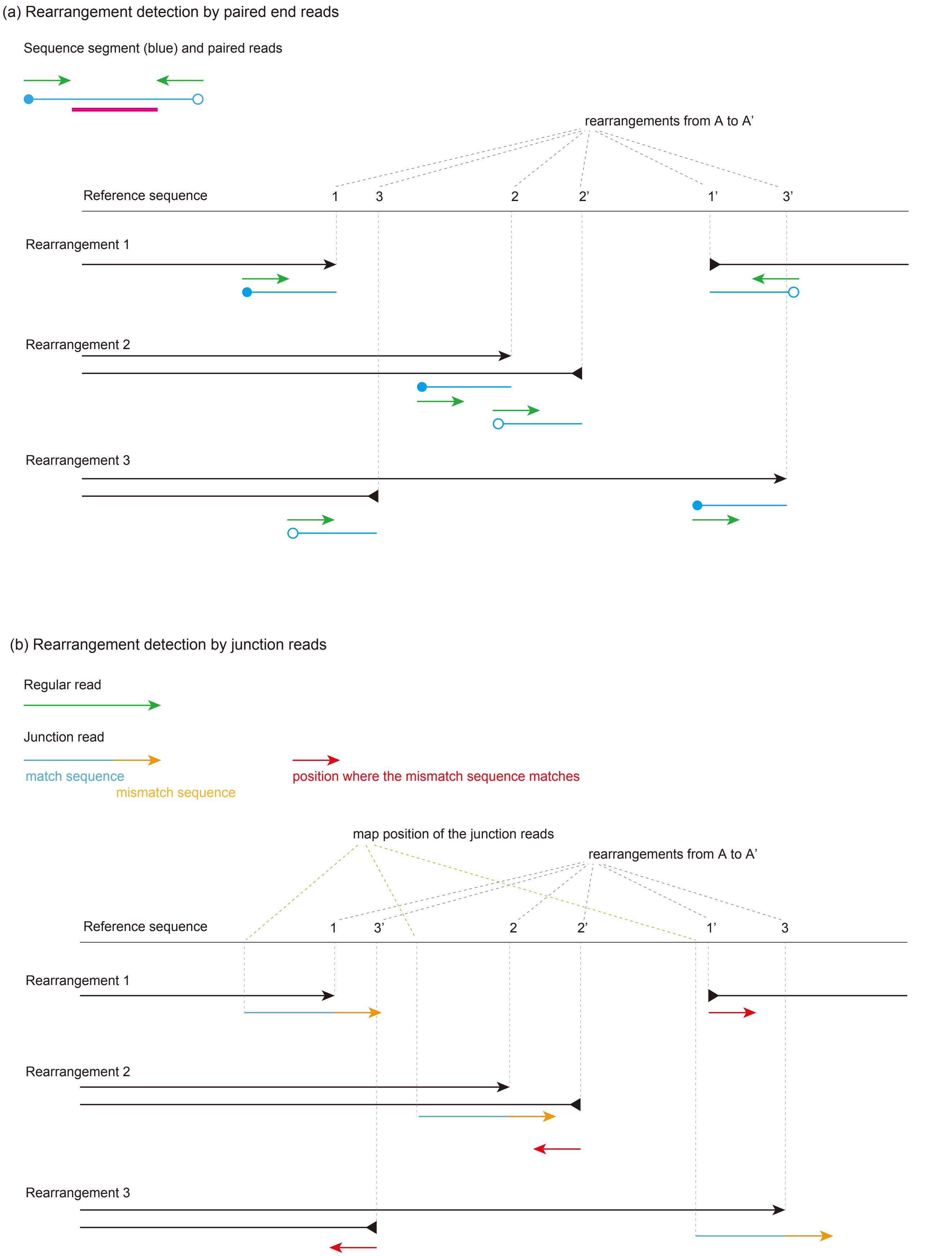

Supplementary Figure 4. Schemes for detection of genomic rearrangements by paired-end reads and junction reads.
a Analysis of genomic rearrangements by mapping of paired-end reads. Paired-end reads mapped to the reference sequences with distant position (Rearrangement 1) or direction (Rearrangement 2) or both (Rearrangement 3).
b Analysis of genomic rearrangements by junction reads. Chimeric reads called as junction reads are extracted, and rearrangements (Rearrangements 1-3) are determined by searching the sequence of the mismatch region.

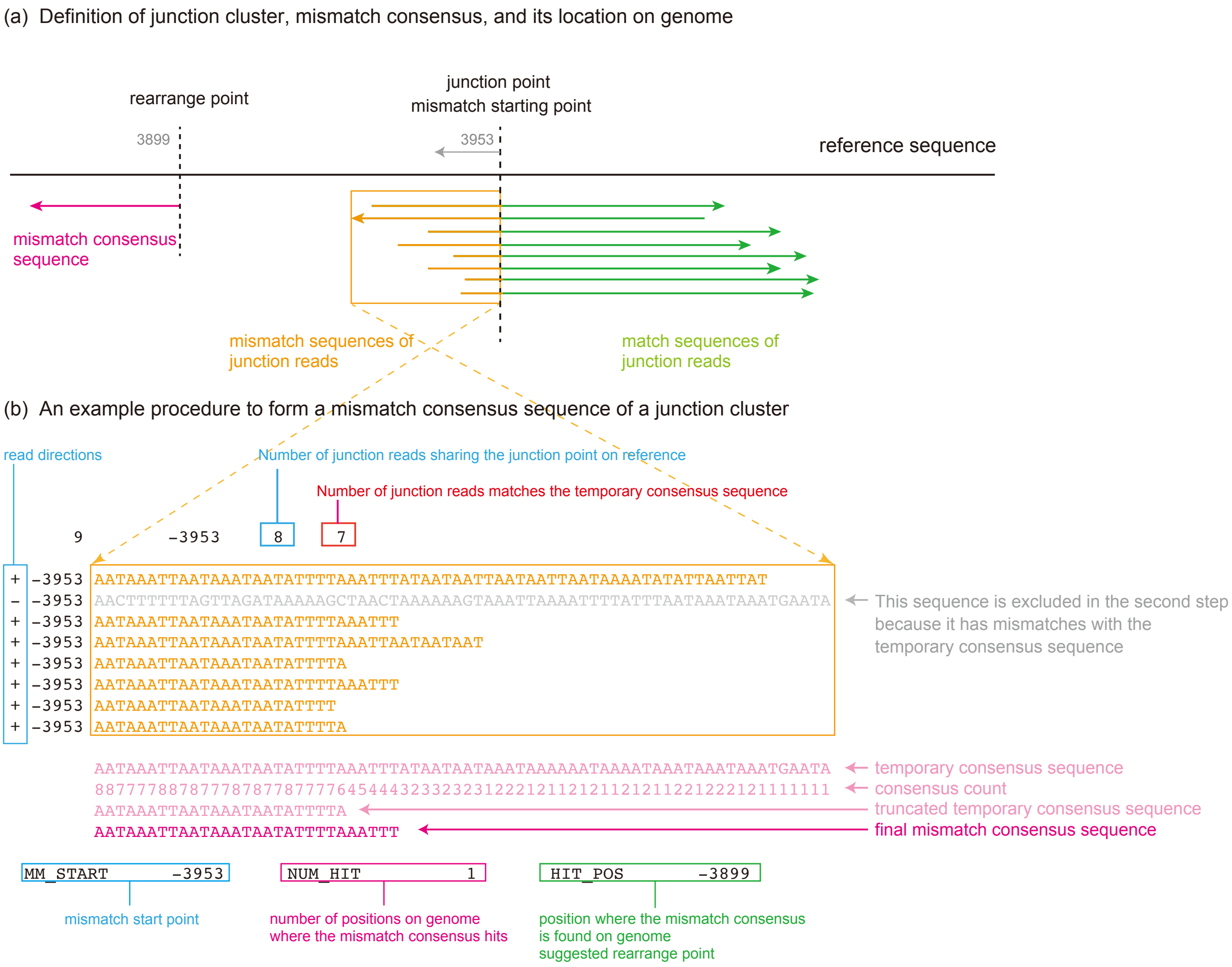

Supplementary Figure 5. Formation of mismatch consensus sequence from a Junction cluster.

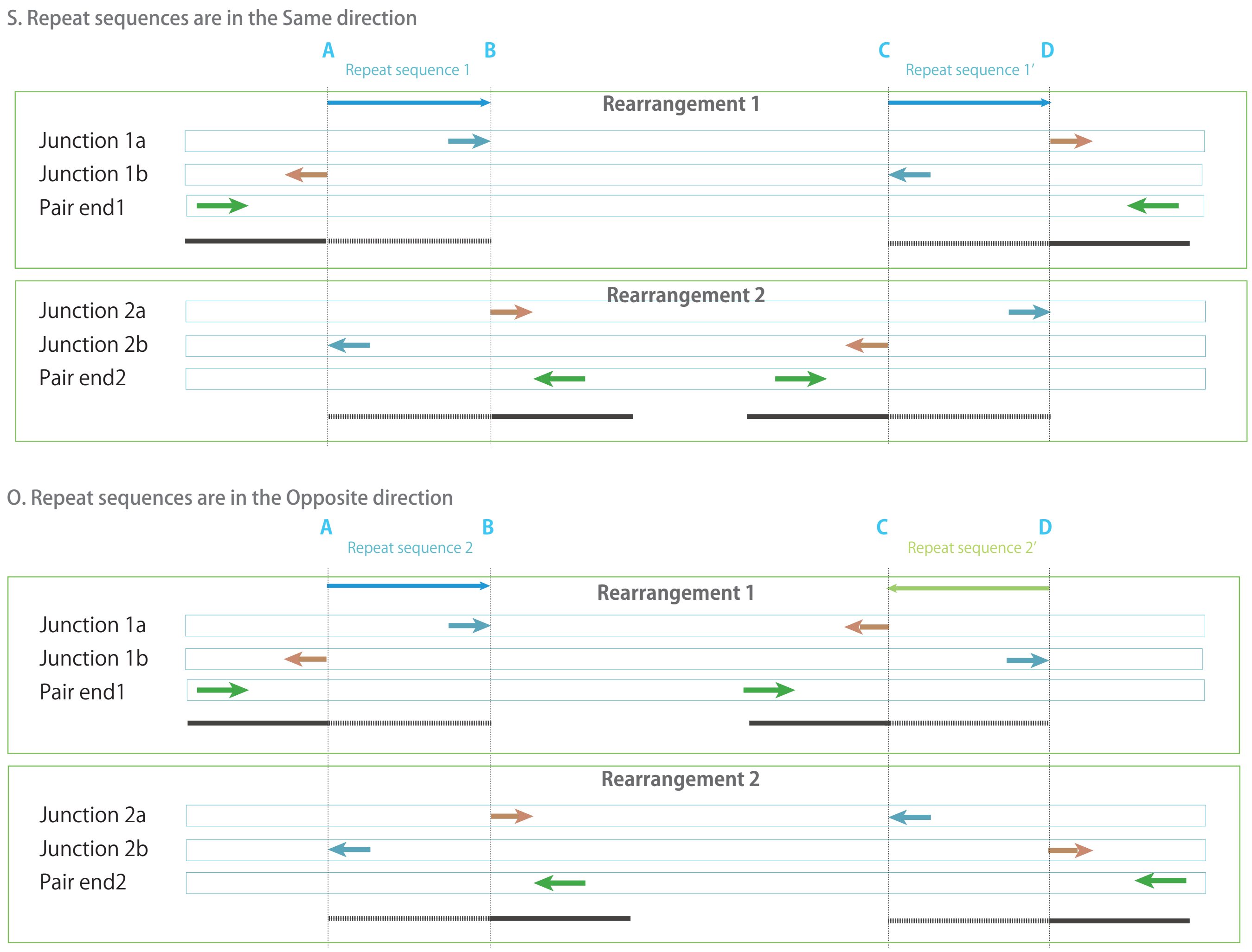

Supplementary Figure 6. Rearrangements with homologous sequences in the same directions (S) and the opposite directions (O).

Two junction clusters (a and b) and a paired end cluster correspond to the same genome rearrangement in a green box. The rearrangement can be described as two black bars connected by overlapping two black dotted bars representing the repeat sequence.

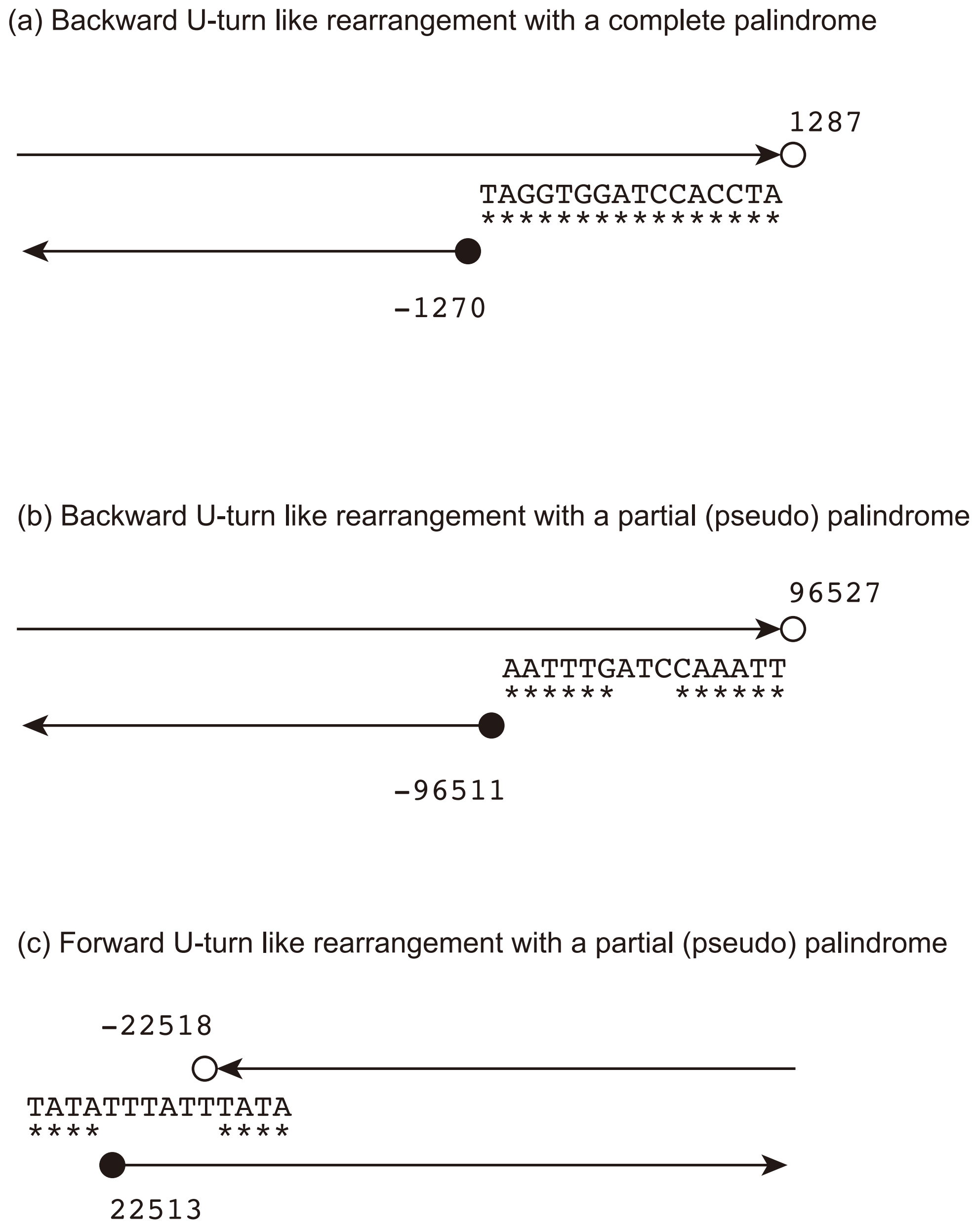

Supplementary Figure 7. Examples of U-turn-like (Palindrome-type) rearrangements.
The position one base before the empty circle, is connected with the base at the filled circle (i.e. the empty and fille circles overlap in the rearranged sequence).

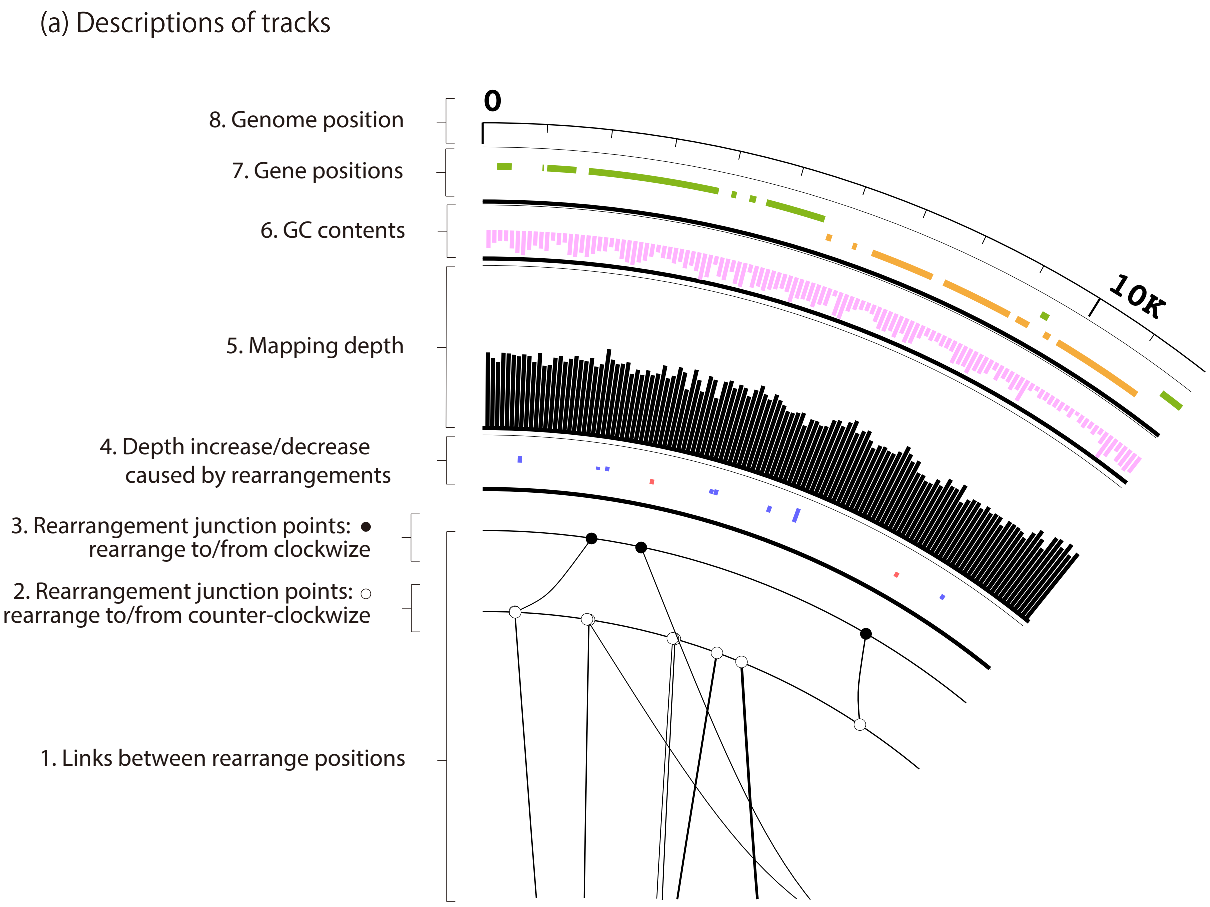

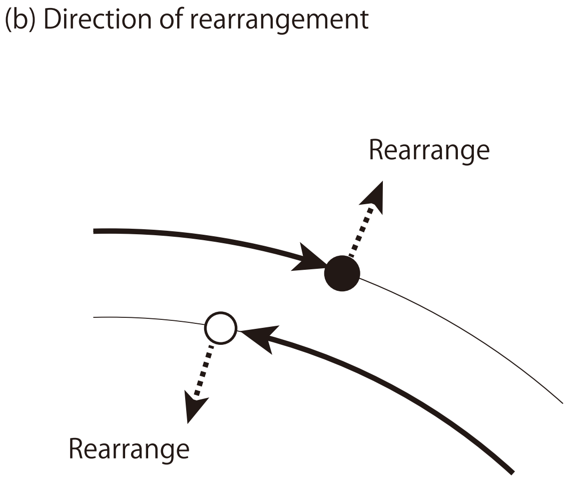

Supplementary Figure 8. Graphical representation of rearrangements.
a. Description of tracks.
b. Direction of the links. See Supplement Method for the detailed description.

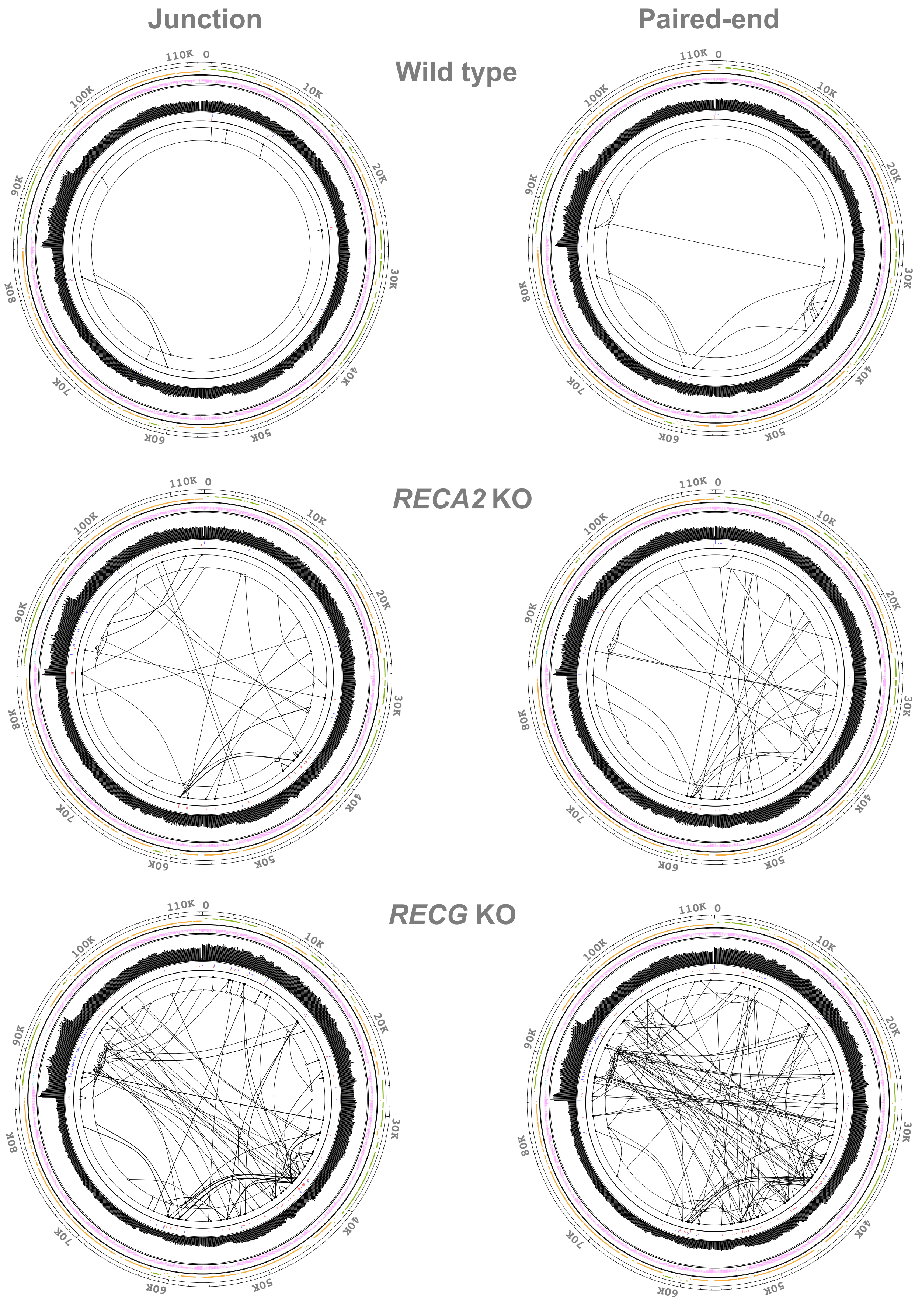

Supplementary Figure 9. Links of junction and paired-end reads in chloroplast DNA.
Rearrangements identified by junction reads and paired-end reads are shown as links on the cpDNA map along with mapping depth. Details of the map are explained in Fig. S8. One copy of the inverted repeats was removed from the maps.

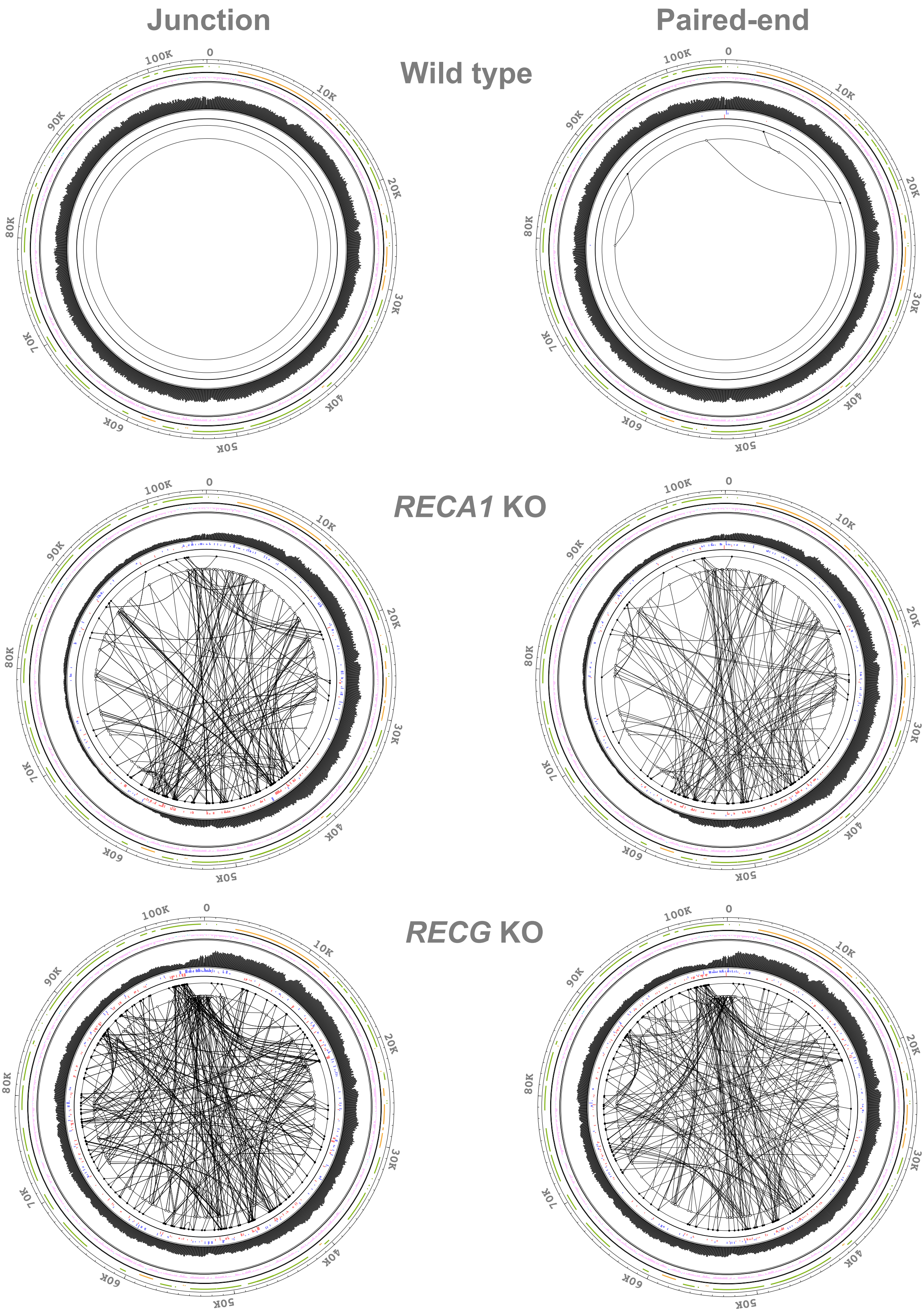

Supplementary Figure 10. Links of junction and paired-end reads in mitochondrial DNA.
Rearrangements identified by junction reads and paired-end reads are shown as links on the mtDNA along with mapping depth. Details of the map are explained in Fig. S8.

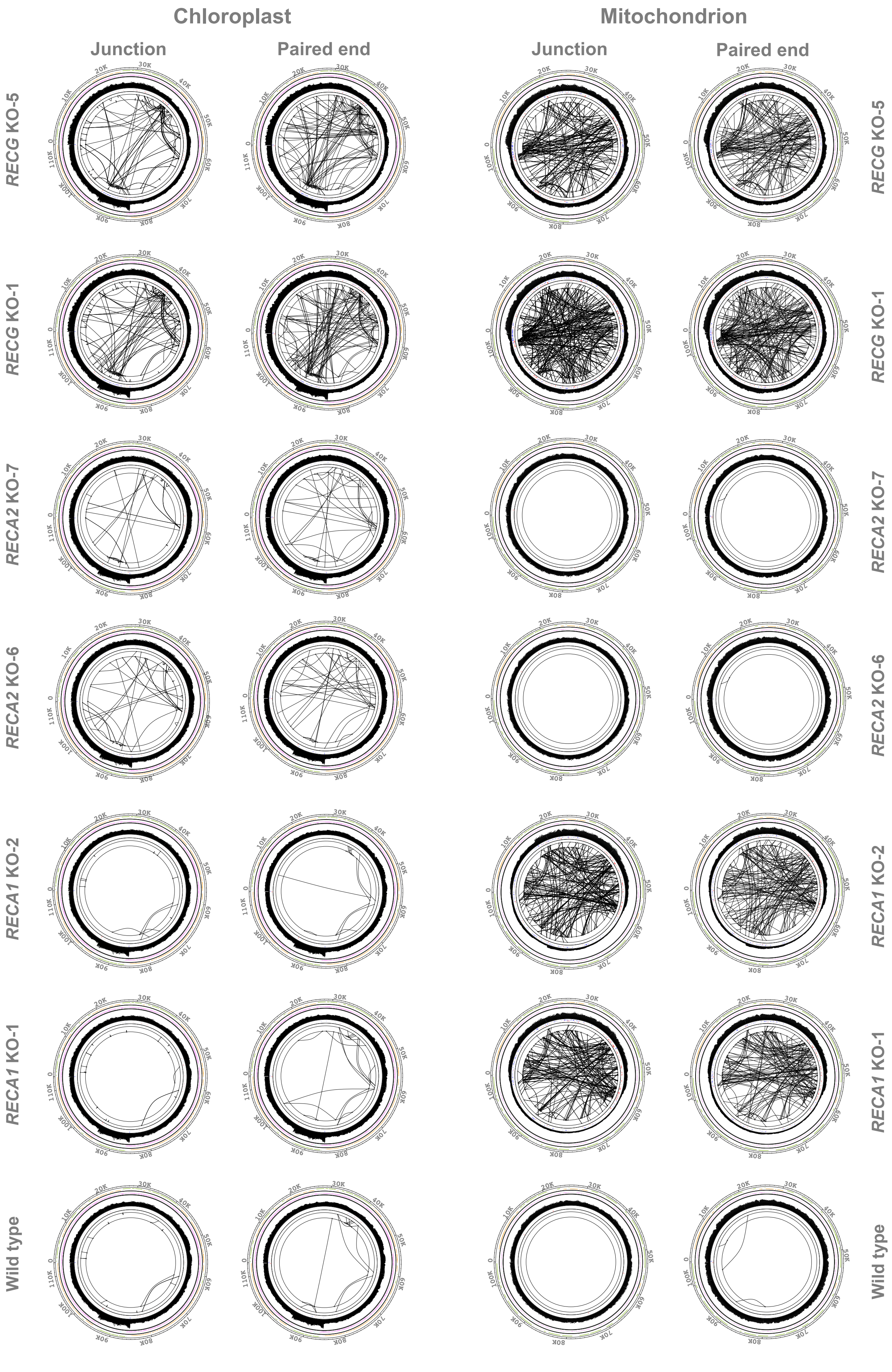

Supplementary Figure 11. Tracks of organelle DNA of all *P. patens* strains.

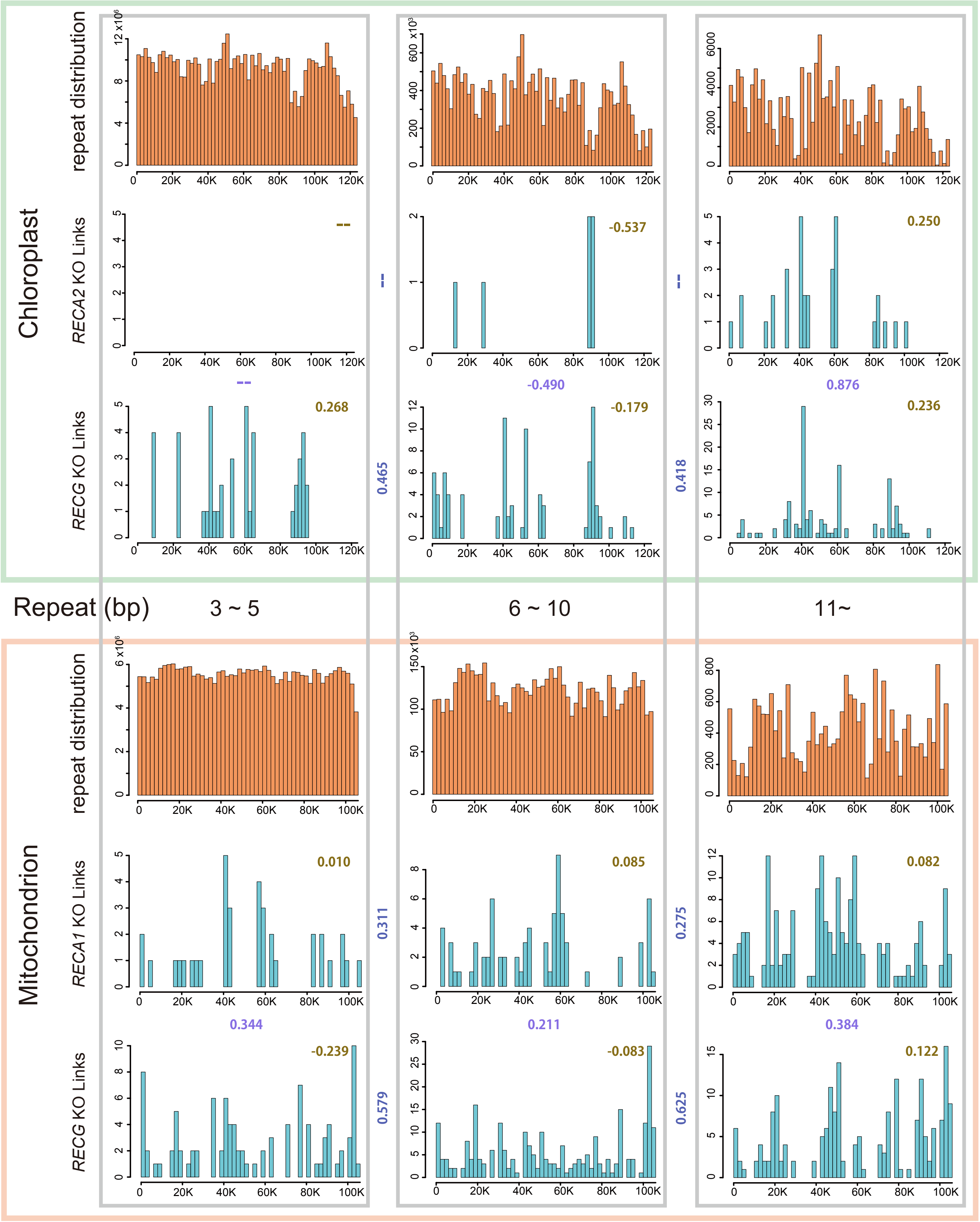

Supplementary Figure 12. Repeat distribution and links of mutant organelle genomes.
The number of repeats in organelle DNA is displayed as histgrams of 2 kb windows (x-axis) along with the organelle map (y-axis). The repeats are categorized by their length, 3~5 bp, 6~10 bp, and 11 bp≤. The links from mutant organelle DNA are similarly categorized and displayed as histograms according to the repeat length found at the junctions. Correlation between each link distribution is shown between the histograms as purple or navy numbers. Correlation between repeat distribution and each of link distributions is shown by brown numbers.

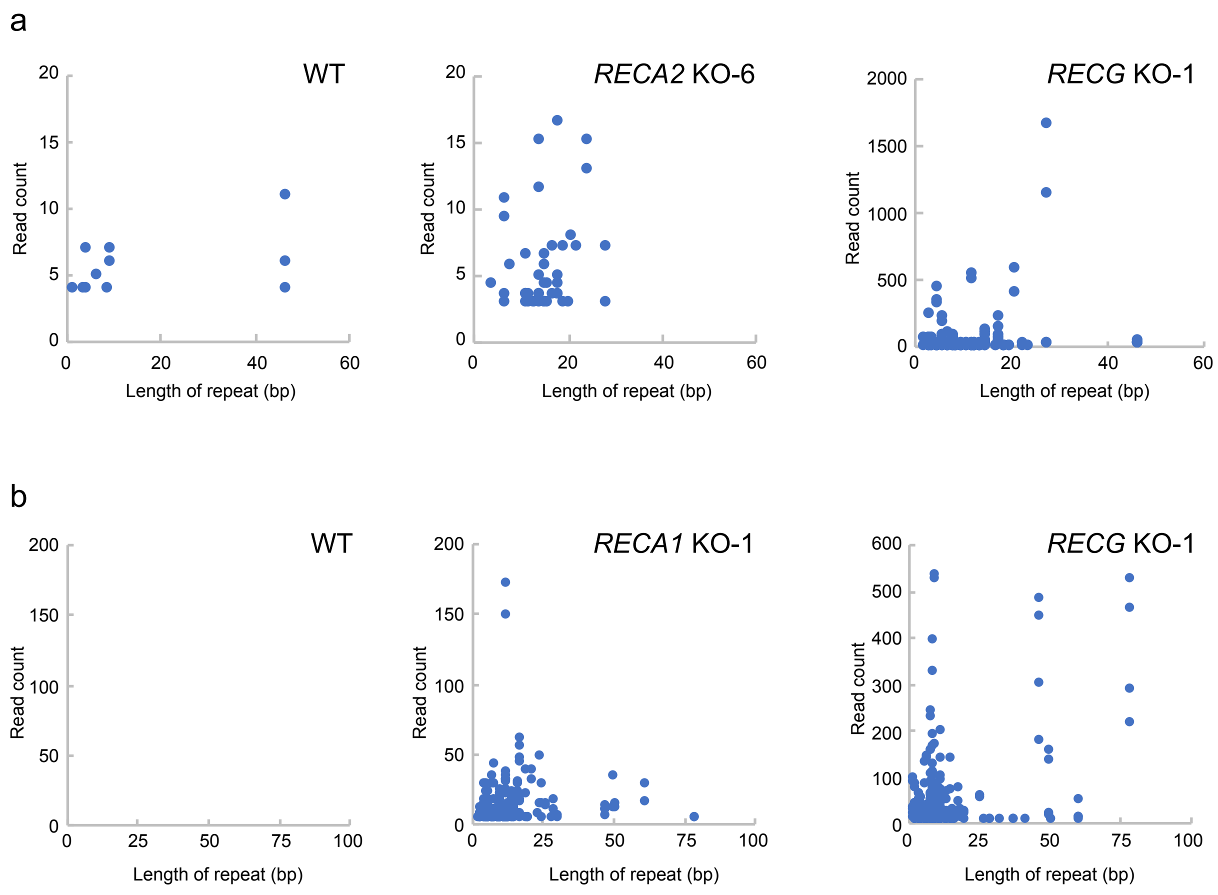

Supplementary Figure 13. Relationship between repeat length and read count.
The length of the repeats and read count of recombination products identified by Junction reads are plotted for chloroplast DNA (**a**) and mitochonddrial DNA (**b**).

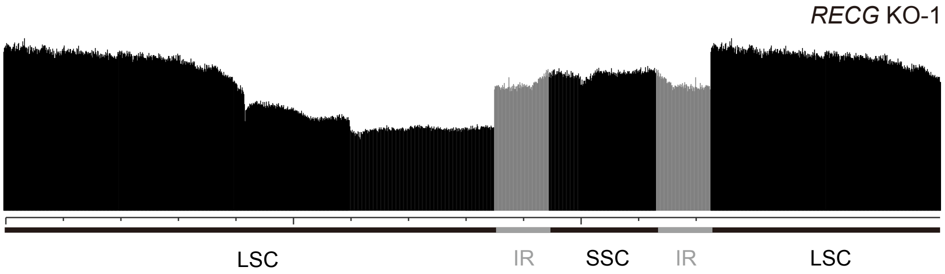

Supplementary Figure 14. Read depth of whole chloroplast DNA of *RECG* KO.
Duplicated IR and LSC of mapped reads of a *RECG* KO mutant are added to the original map.

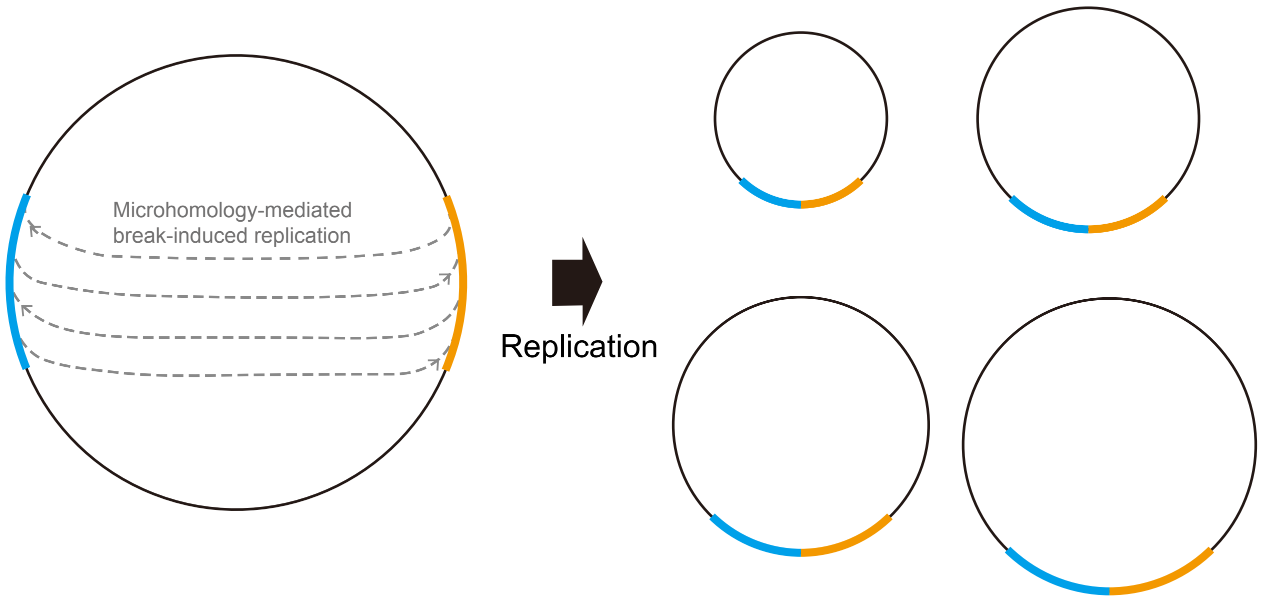

Supplementary Figure 15. A model for recombination-driven depth variation of mutant organelle genomes. In the mutant, replication stall or collapse produces single DNA end that induce microhomology-mediated replication between specific areas colored in blue and orange, thereby generating subgenomes.

Supplementary Table 1. Sequencing results of *P. patens* strains

Strains	Number of read	Total read bases (bp)	Read length (bp)	Q30	GC
wild type	67,634,898	10,145,234,700	150	88.55%	32.00%
RECA1 KO-1	64,220,846	9,633,126,900	150	88.12%	31.72%
RECA1 KO-2	70,062,734	10,509,410,100	150	88.24%	31.82%
RECA2 KO-6	71,158,278	10,673,741,700	150	87.71%	32.51%
RECA2 KO-7	62,269,830	9,340,474,500	150	88.14%	32.55%
RECG KO-1	67,588,198	10,138,229,700	150	88.31%	31.47%
RECG KO-5	74,366,922	11,155,038,300	150	88.30%	31.34%

Supplementary Table 2. Number of mapped reads by mismatch tolerant (h75r10), less tolerant (h2r3), or truncated (chp100_h2r3) condition

	Number of reads from experimen t	Number of reads mapped on chromosomes						Number of reads mapped on chloroplast genome						Number of reads mapped on mitochondrial genome						Total number of mapped reads					
		h75r10		h2r3		chp100_h2r3		h75r10		h2r3		chp100_h2r3		H75r10		h2r3		chp100_h2r3		h75r10		h2r3		chp100_h2r2	
WT	67634898	42658482	63.1%	37211186	55.0%	39903478	59.0%	21975239	32.5%	19052459	28.2%	20658413	30.5%	2728553	4.0%	2392799	3.5%	2566448	3.8%	67362274	99.6%	58656444	86.7%	63128339	93.3%
RECA1 KO-1	64220846	37502198	58.4%	32532561	50.7%	34966090	54.4%	23741606	37.0%	20475799	31.9%	22255581	34.7%	2724281	4.2%	2370499	3.7%	2550861	4.0%	63968085	99.6%	55378859	86.2%	59772632	93.1%
RECA1 KO-2	70062734	40703231	58.1%	35419127	50.6%	38001820	54.2%	25488635	36.4%	22027709	31.4%	23910435	34.1%	3613525	5.2%	3155917	4.5%	3388278	4.8%	69805391	99.6%	60602753	86.5%	65300533	93.2%
RECA2 KO-6	71158278	52680190	74.0%	45393378	63.8%	48920485	68.7%	15956147	22.4%	13662078	19.2%	14890554	20.9%	2195140	3.1%	1895629	2.7%	2046011	2.9%	70831477	99.5%	60951085	85.7%	65857050	93.6%
RECA2 KO-7	62269830	46200645	74.2%	40054434	64.3%	43058934	69.1%	13567833	21.8%	11689340	18.8%	12701097	20.4%	2246098	3.6%	1954237	3.1%	2102333	3.4%	62014576	99.6%	53698011	86.2%	57862364	92.9%
RECG KO-1	67588198	32815785	48.6%	28505278	42.2%	30624625	45.3%	29618166	43.8%	25593634	37.9%	27804415	41.1%	4895779	7.2%	4273188	6.3%	4594809	6.8%	67329730	99.6%	58372100	86.4%	63023849	93.2%
RECG KO-5	74366922	35716931	48.0%	31036159	41.7%	33332012	44.8%	33152947	44.6%	28684395	38.6%	31132405	41.9%	5194549	7.0%	4526259	6.1%	4871108	6.6%	74064427	99.6%	64246813	86.4%	69335525	93.2%

Supplementary Table 3. Correlation coefficient between mapping depth of WT and mutants

		Correlation coefficient
Chloroplast	WT - *RECA1* KO-1	0.998
	WT - *RECA1* KO-2	0.997
	WT - *RECA2* KO-6	0.928
	WT - *RECA2* KO-7	0.911
	WT - *RECG* KO-1	0.667
	WT - *RECG* KO-5	0.696
Mitochondrion	WT - *RECA1* KO-1	0.255
	WT - *RECA1* KO-2	0.191
	WT - *RECA2* KO-6	0.903
	WT - *RECA2* KO-7	0.910
	WT - *RECG* KO-1	0.384
	WT - *RECG* KO-5	0.423

Supplementary Table 4. Mapping ratio of all junction reads, and their pair reads (Number of matching bases / Length of mapped reads including mismatches (Mapping ratio)). Mapping of junction reads is rather strict compared to regular reads. Two ends of the junction reads are mapped on separate positions on reference genome. The longer side of the junction reads are allowed no mismatch (100% mapping ratio) and the shorted side is allowed one mismatch maximum for a read (>99.7% mapping ratio). Overall, the matching ratio of junction reads are quite high (>99.9%). On the other hand, a pair read of a junction read is mapped on one position, even if it includes junction point inside. Therefore, the mapping ratio is relatively low (>96.5%).

		Junction long side	Junction short side	Junction total	Pair reads of Junction reads	Total
Chloroplast	*RECA1* KO-1	422,895 / 422,895 (100.00)	139,993 / 140,355 (99.74)	562,888 / 563,250 (99.94)	552,395 / 563,250 (98.07)	1,115,283 / 1,126,500 (99.00)
	RECA1 KO-2	502,784 / 502,748 (100.00)	166,388 / 166,852 (99.72)	669,136 / 669,600 (99.93)	656,246 / 669,600 (98.01)	1,325,382 / 1,339,200 (98.97)
	RECA2 KO-6	286,455 / 286,445 (100.00)	94,481 / 94,695 (99.77)	380,936 / 381,150 (99.94)	374,217 / 381,150 (98.18)	755,153 / 762,300 (99.06)
	RECA2 KO-7	285,049 / 285,049 (100.00)	92,435 / 92,651 (99.77)	377,484 / 377,700 (99.94)	370,939 / 377,700 (98.21)	748,423 / 755,400 (99.08)
	RECG KO-1	1,629,017 / 1,629,017 (100.00)	525,049 / 526,033 (99.81)	2,154,066 / 2,155,050 (99.95)	2,109,139 / 2,155,050 (97.87)	4,263,205 / 4,310,100 (98.91)
	RECG KO-5	1,872,570 / 1,872,570 (100.00)	607,182 / 608,280 (99.82)	2,479,752 / 2,480,850 (99.96)	2,435,049 / 2,480,850 (98.15)	4,914,801 / 4,961,700 (99.05)
	WT	425,142 / 425,142 (100.00)	141,769 / 142,158 (99.73)	566,911 / 567,300 (99.93)	554,858 / 567,300 (97.81)	1,121,769 / 1,134,600 (99.01)
Mitochondrion	*RECA1* KO-1	396,819 / 396,819 (100.00)	134,118 / 134,331 (99.84)	530,937 / 531,150 (99.96)	523,066 / 531,150 (98.48)	1,054,003 / 1,062,300 (99.22)
	RECA1 KO-2	763,615 / 763,615 (100.00)	255,417 / 255,417 (99.86)	1,019,032 / 1,019,400 (99.96)	1,003,548 / 1,019,400 (98.45)	2,022,580 / 2,038,800 (99.20)
	RECA2 KO-6	5,725 / 5,725 (100.00)	1,923 / 1,925 (99.90)	7,648 / 7,650 (99.97)	7,353 / 7,650 (96.12)	15,001 / 15,300 (98.05)
	RECA2 KO-7	10,495 / 10,495 (100.00)	3,449 / 3,455 (99.83)	13,944 / 13,950 (99.96)	13,468 / 13,950 (96.55)	27,412 / 27,900 (98.25)
	RECG KO-1	1,129,271 / 1,129,271 (100.00)	354,863 / 355,429 (99.84)	1,484,134 / 1,484,700 (99.96)	1,455,872 / 1,484,700 (98.06)	2,940,006 / 2,969,400 (99.01)
	RECG KO-5	1,286,725 / 1,286,725 (100.00)	390,139 / 390,725 (99.85)	1,676,864 / 1,677,450 (99.97)	1,642,982 / 1,677,450 (97.95)	3,319,846 / 3,354,900 (98.96)
	WT	12,748 /12,748 (100.00)	4,343 / 4,352 (99.79)	17,091 / 17,100 (99.95)	16,556 / 17,100 (96.82)	33,647 / 34,200 (98.38)

Supplementary Table 5. Number of reads found in paired read clusters and junction read clusters corresponding to some rearrangements caused by homology or microhomology as examples.

	[1]Direction of repeat	[2]Junction 1	Junction 2	Homologous sequence	Paired read 1	Paired read 2
WT CP	O	61,657 / -80,856 (4)<br>80,904 / -61,609 (6)	----<br>-61,609 / 80,904 (11)	TTTTGAAATAGAGGCGATAATATAATAAAAACGTCGACTATAACCCT	80,764 / 61,415 (14)	-61,668 / -80,956 (17)
RECA2 KO-7 CP	S		-6,154 / -59,122 (43)<br>59,142 / 6,174 (43)	CTGCCTTACCACTTGGCCA		-6,250 / 58,941 (72)
RECG KO-1 CP	S	----<br>-38,897 / -36,673(13)	----<br>-36,673 / -38,897(10)	CATATGTATAGAACTAATTTTGG	36,503 / -38,941 (81)	38,854 / -36,856 (35)
RECG KO-5 CP	S	17,822 / 94,929 (267)<br>-94,918 / -17,811 (296)		TGGATTTGGT	17,644 / -94,998 (379)	
RECA1 KO-1 MT	S	40,872 / 92,752 (11)<br>-92,739 / -40,859 (9)		GGAAAAAAGGGC	40,683/ -92,831 (18)	
RECA1 KO-1 MT	S	41,739 / 44,212 (172)<br>-44,199 / -41,726 (150)		CATTTGGTCGAC	41,549 / -44,291 (255)	
RECG KO-1 MT	S	201,25 / 102,409 (112)<br>-102,361 / -20,077 (249)	102,409 / 20,125 (170)<br>-20,077 / -102,361 (100)	TGAGAAGAGCCGTATGAGGCCGTAGGCTCACGTACGGTTCGGAAGCC	-102,457 / 19,931 (612)	102,243 / -20,194 (309)<br>-20,528 / 102,731 (100)
RECG KO-5 MT	S		50,213 / 612 (150)<br>-605 / -50,206 (157)	AGTGAT		-672 / 50,018 (182)

[1]S, same direction; O, opposite direction, see Figure S6.

[2]Positions of the junctions are shown by position-1 / position-2 with their read number in parentheses.

Supplementary Table 6. Number of repeats (≥15 bp) in chloroplast and mitochondrial DNA

	Number of repeats	
Length of repeat	Chloroplast DNA	Mitochondrial DNA
>1000 bp	2	0
100-1000 bp	0	0
50-100 bp	5 (0)	5 (5)
30-49 bp	8 (1)	12 (5)
20-29 bp	41 (29)	119 (93)
15-19 bp	2075 (2048)	773 (750)

Repeats were identified in organelle DNA by REPuter [1] with a parameter (≥15 bp, no mismatch).

Numbers in parentheses denote number of repeats except for palindromic sequences.

Supplementary Table 7. Numbers of junction reads and junction clusters

Strain	Number of reads	Chloroplast		Mitochondrion	
		Number of junction reads	Number of junction clusters	Number of junction reads	Number of junction clusters
WT	67634898	657686	326	59598	8
RECA1 KO-1	64220846	719102	313	66034	176
RECA1 KO-2	70062734	760998	346	86185	220
RECA2 KO-6	71158278	492830	280	50753	4
RECA2 KO-7	62269830	409230	259	50530	5
RECG KO-1	67588198	922543	511	120781	326
RECG KO-5	74366922	1009378	498	128790	221

Supplementary Table 8. Number of rearrangements detected by paired end (PE) clusters and pairs (in parentheses) compared with the corresponding rearrangements detected by the junction cluster (JC) method

		WT	*RECA1* KO-1	*RECA1* KO -2	*RECA2* KO -6	*RECA2* KO -7	*RECG* KO -1	*RECG* KO -5
Chloroplast	PE	15 (160)	19 (174)	11 (95)	40 (387)	35 (373)	130 (4,409)	117 (4,838)
	JC	9 (83)	8 (74)	10 (48)	31 (329)	27 (356)	126 (8,196)	101 (9,148)
Mitochondrion	PE	4 (49)	146 (4,003)	152 (5,981)	1 (7)	2 (27)	202 (8,137)	136 (9,227)
	JC	0 (0)	160 (3,097)	188 (6,230)	0 (0)	0 (0)	232 (9,401)	140 (10,361)

The numbers of rearrangements are smaller than the numbers of the junction clusters (mhr in Table 2), because each rearrangement consists of a pair of junction clusters. We often find only one cluster for a pair, and therefor the number of the rearrangement is more than half of the number of junction cluster.

Supplementary Table 9. Evaluation of previously identified recombination products in mtDNA and cpDNA

	Junction read		Previous data			
Repeat #	*RECA1* KO	*RECG* KO	*RECA1* KO	*RECG* KO	Method	References
Mitochondrial recombination products						
R1	+	++	+	+/++	gel blot	Odahara et al. (2015)
R2	+/++	+	+	not tested	gel blot	Odahara et al. (2009)
R3	+	+	+	not tested	gel blot	Odahara et al. (2009)
R4	+	-[a]	+	-	gel blot	Odahara et al. (2015)
R5	+/-	-	++	+	qPCR	Odahara et al. (2015)
R6	+	-	+	-	gel blot	Odahara et al. (2015)
R9	+	++	-	+	gel blot	Odahara et al. (2015)
R10	+	-	+	-	gel blot	Odahara et al. (2015)
R11	-	-/+	+/++	+	qPCR	Odahara et al. (2015)
R12	-	-/+	+	+	qPCR	Odahara et al. (2015)
R13	+	-	+/++	+	qPCR	Odahara et al. (2015)
R15	-	-	+	+/++	qPCR	Odahara et al. (2015)
R18	-	-	+/++	+	qPCR	Odahara et al. (2015)
R19	-	-	+	+	qPCR	Odahara et al. (2015)

Chloroplast recombination products

Repeat #	*RECA2* KO	*RECG* KO	*RECA2* KO	*RECG* KO		
IR-1	-	+	+	++	qPCR	Odahara et al. (2017)
DR-1	-/+	+	+	+	qPCR	Odahara et al. (2017)

[a]-, not detected in the assay.

Supplementary Table 10. Morisita's *Iδ*-index[2] for the distribution of links.

	Chloroplast			Mitochondrion		
**window size**	100	1,000	10,000	100	1,000	10,000
**WT**	174.4/252.0	17.5/25.3	2.9/3.6	---/---	---/---	---/---
***RECA1* KO-1**	226.0/283.0	22.8/28.5	3.8/7.5	20.8/18.8	5.1/3.7	3.2/1.6
***RECA1* KO-2**	---/189.0	---/19.0	4.0/4.5	20.4/15.6	4.7/3.0	2.8/1.4
***RECA2* KO-6**	155.9/61.9	13.4/8.7	2.6/2.0	---/---	---/---	---/---
***RECA2* KO-7**	137.5/77.8	13.4/11.3	2.7/2.5	---/---	---/---	---/---
***RECG* KO-1**	39.6/26.0	8.1/5.9	2.3/2.2	10.0/10.7	2.9/3.9	1.2/1.3
***RECG* KO-5**	41.2/30.7	10.5/7.8	2.6/3.2	12.9/13.3	3.1/4.4	1.4/1.3

Numbers larger than 1.0 indicates the distribution is uneven and concentrated in specific regions. Numbers on left in each cell are for links toward the upstream and numbers on right are for links toward downstream.

Supplementary Table 11. List of mutations found and modified between the reference downloaded from the public database and our strain. The database ID of reference sequences of *Physcomitrella patens*, are NC_007945.1 for mitochondrion, and NC_005087.2 for chloroplast. Mutations clustered in close vicinity are highlighted in yellow.

Mitochondrion: NC_007945.1

	Mutation type	Position	from	to
1	SNP	48612	N	T
2	SNP	50872	Y	T

Chloroplast: NC_005087.1

	Mutation type	Position	from	to
1	DEL	9798	G	-
2	DEL	9799	T	-
3	SNP	9800	G	A
4	SNP	9802	T	C
5	SNP	11087	G	A
6	SNP	12135	C	A
7	INS	12312	-	A
8	SNP	15520	C	T
9	DEL	16622	T	-
10	SNP	16865	C	A
11	INS	18656	-	T
12	SNP	19321	T	C
13	SNP	25847	A	G
14	SNP	25854	G	T
15	SNP	25855	G	T
16	SNP	25856	C	T
17	INS	26158	-	A
18	DEL	28539	A	-
19	SNP	29840	C	T
20	DEL	32408	A	-
21	SNP	36121	A	G
22	SNP	38030	G	A
23	SNP	38064	G	A
24	SNP	38134	C	T
25	DEL	41964	A	-
26	SNP	44958	T	A
27	SNP	44960	T	A
28	SNP	44965	T	A
29	SNP	44970	T	A
30	INS	44980	-	A
31	INS	45033	-	T
32	SNP	45236	G	A
33	SNP	46444	G	A
34	INS	52816	-	T
35	INS	54587	-	T
36	INS	56466	-	A

37	INS	56497	-	A
38	INS	56499	-	T
39	INS	56501	-	A
40	INS	56507	-	A
41	SNP	56509	G	A
42	SNP	56812	A	T
43	INS	56899	-	T
44	INS	56977	-	T
45	INS	57138	-	A
46	INS	60800	-	T
47	SNP	60921	G	A
48	SNP	61320	G	A
49	SNP	64077	G	C
50	SNP	64108	G	C
51	DEL	65067	T	-
52	DEL	65068	T	-
53	INS	65075	-	A
54	SNP	66271	A	T
55	SNP	67289	G	A
56	SNP	68420	A	G
57	SNP	69775	T	A
58	SNP	70238	T	A
59	SNP	70648	G	A
60	INS	93772	-	T
61	SNP	96104	C	A
62	SNP	97891	C	A
63	INS	98104	-	AAA
64	SNP	98858	C	T
65	SNP	107202	C	T
	DEL	113302~122890		

Supplementary References

1. Kurtz S, Choudhuri JV, Ohlebusch E, Schleiermacher C, Stoye J, Giegerich R. REPuter: the manifold applications of repeat analysis on a genomic scale. *Nucleic Acids Res* **29**, 4633-4642 (2001).

2. Morisita M. I σ -Index, a measure of dispersion of individuals. *Researches on Population Ecology* **4**, 1-7 (1962).

Procedure to perform mapping and following analysis of rearrangements in organelle

This procedure includes all the programs used to carry out the analysis in the papre.

"Ultra-deep sequencing reveals dramatic alteration of organellar genomes in Physcomitrella patens due to biased asymmetric recombination"

by Odahara, Nakamura, Oshima and Sekine.

The file suffix appears in the paper .mhr and .mhmr are replaced with .hr and .hmr, respectively in this document and with the actual programs.
Likewise, program names in the paper such as comp_mhr and comp_mhmr are replaced with comp_hr and comp_hmr, respectively.

It includes many analysis.
The main part of the rearrangement analysis including the drawing of links, can be carried out through the section 0.0-3.2.

Lines with '%' in the first column is the command line to input,
and /DBdir/ is the directory you put the reference files.

----

0. Preparing the mapping: Indexing and Reference list

0.0 Indexing reference sequence (mkindex)

Our mapping software immap uses simple fixed length index.
To make the index, use 'mkindex' with specified index length.
For instance, to make an index file for the reference file 'foo.fasta' with index length 14, type

```
% mkindex -14 foo.fasta
```

An index file 'foo.fasta.index14' will be created.
The index file should stay in the same directory as the reference file (foo.fasta).
The most appropreate index length depends on the length of reference (10~15).

0.1 Prepareing reference list file (ref.fasta)

Our mapping software immap only takes single fasta files as input.
When the reference sequence consists of multiple sequence,
such as in the case of multiple chromosomes of eukaryotic genomes,
we need to prepare a list file of single fasta files as follows.

```
========================= ref.list ============================
#Title line
0       /DBdir/CH_MOD.fasta                  /DBdir/CH_MOD.gb
0       /DBdir/MT_MOD.fasta                  /DBdir/MT_MOD.gb
0       /DBdir/Ppatens.fa.out.fasta
===============================================================
```

The first line starts with # and is a title line.
Following lines starts with 0, and are followed by one or two space separated colums.
The first column is the reference single fasta file name, and the second column is the Genbank format file that includes the description/annotation of the reference file.
The genbank file (.gb) may be omitted.
In our analysis, the first 2 files are for the genome sequences of organelles (chroloplast and mitochondorion),
and the third file contains nuclear chromosome sequences.
The multiple nuclear choromosome sequences are connected with 500 N's as linkers to form a single fasta format.
The index files for each fasta files should be prepared according to the procedure 0.0 prior to the mapping.

----

1 Mapping (immap, chopfastq)

1.1 Mapping original read (100~300bp) allowing many mismatches to detect junction reads

To carry out the mapping, use immap as follows.

```
% immap -a 16 -i 14 -h 75 -r 10 -job i14h75r10 ref.list PpWT.fastq
```

Here the last argument (PpWT.fastq) is the read file,
and the second last argument (ref.list) is the list of reference fasta files described in the previous section(0.1).
Paired end reads should be merged into a single file in the order of R1-R2.
Following is the description of options with '-'.

```
-a   # :  Number of threads for multi thread calculations.
-i   # :  Index length. An appropreate index file should be prepared according to 0.0
-h   # :  Maximum number of mismatch per read. The value should be as many as half the
```

```
          length of reads for junction analysis.
-r   # :  Number of search per read shifting index position. h+1 if possible.
          10~20 if h is large.
-job ~ :  Job name. We usually put the mapping parameters as shown in the example.
```

The result of mapping is output in the .hit file. .hit file consists of 2 or 3 columns.
The first column is the read id. (sequentical number), and the second column is the mapped position of the read.
The negative value for the mapped position indicates the read is mapped as reverse complement, and the absolute value indicate the mapped position of the 5' end of the reverse complement read on the reference.
The third column (if exist) is the mapping depth which can be used to display the read alignment.

immap generates .hit output file for each reference file in the reference list file with index number starting from 0.
For instance, there are 3 reference files (CH_MOD.fasta, MT_MOD.fasta and Ppatens.fa.out.fasta) in the ref.list described in 0.1.
In this example immap outputs, PpWT.fastq_i14h75r10_0.hit for the hits on the first reference CH_MOD.fasta, and PpWT.fastq_i14h75r10_1.hit for the hits on the second reference MT_MOD.fasta.

By adding -map option, immap generate a text file (.map) that shows the mapping status of each read.
The width of .map file is 300 by default and can be altered with -mw option.
Add option '-mw 100' to make the width of the map file 100.

1.2 Mapping of truncated reads (chopfastq)

In our study, 150bp reads were used for the junction read analysis.
Besides, reads truncated to 50bp were used for the analysis of mapping depth, and rearrangement analysis using paired end reads.
To prepare the truncated reads, we used 'chopfastq'.

```
% chopfastq -l 50 PpWT.fastq
```

This generates chp50_PpWT.fastq that contains truncated reads.
The generated reads are mapped using immap allowing upto 2 bp mismatch.

```
% immap -a 16 -i 14 -h 2 -r 3 -job i14h2r3  ref.list chp50_PpWT.fastq
```

Options and arguments are as same as the example in 1.0.

---

2   Depth plot, and preparation of the .peb file used in circos-like plot
    described later(2,3) (ispmap) Depth plot with calibration will be described in 4.0.

2.1 Generage depth plot (histogram) and generate '.peb' file for circmaps

To generate a postscript file describing mapping depth, and to generate .peb files that contains mapped read population along the genome sequence, used for the circos-like plot (3) and calibrated depth plot (4), we use 'ispmap' as follows.

```
% ispmap -peb 1 -hd 50 -ds 0.02 -job i14h2r3_0 /DBdir/CH_MOD.fasta chp50_PpWT.fastq
% ispmap -peb 1 -hd 50 -ds 0.02 -job i14h2r3_1 /DBdir/MT_MOD.fasta chp50_PpWT.fastq
```

The last two arguments are the reference file and read file.
Unlike 'immap', the reference file is specified as a single fasta file, not the list of reference files.
In our example ispmap is carried out for both chroloplast (CH_MOD.fasta) and mitochondorion (MT_MOD.fasta).
Please note that the suffix of the job names (string after -job) are '_0' for chroloplast and '_1' for mitochondorion as these suffix are applied by immap for each '.hit' file.

As of other options, '-peb 1' specifies the generation of read population for every '1' base of reference, that is used for circos-like plot.

Options '-hd 50' and '-ds 0.02' are used for the postscript depth display from ispmap and does not affect .peb file for circos-like plot.

```
-hd   #   : Window size of depth  depth count along reference sequence
-ds   #.# : Scale factor of the Y axis (depth) of the postscript plot
```

2.2 Generate base resolution mapping plot and text .map file.

The former example generates depth histogram output.
Another option can display each reads mapped underneath the reference sequence, showing the mismatch base as red dots. To generate this type of output, use '-bw 1' and '-cc' instead of '-hd 50' and '-ds 0.02'.

```
% ispmap -bw 1 -cc -map -job i14h75r10_0 /DBdir/CH_MOD.fasta PpWT.fastq
```

.map file can be generated with ispmap, if you forgot to make it with immap.
'-cc' is a coloring option (no other choice available at the moment).

```
Please note the output postscript file by -bw 1 option can be quite large,
if the reference sequence is very long.

-------------------------------------------------------------------------------------------
-------------------------------------------------------------------------------------------

3   Rearrangement analysis and visualization
    Paired-end read analysis, Junction read analysis, and Generation of Circos-like map.
    (midhr, circmaps, idss, exclude_id_pairs, sort_pdist)

-------------------------------------------------------------------------------------------

3.1.0 Paired-end read analysis  (midhr, idss, exclude_id_pairs, sort_pdist, circmaps)

To perform rearrangement analysis using 'midhr' we use the mapping results
of truncated reads (2.1).

% midhr -ptd 1000 -job i14h3r4_0 /DBdir/CH_MOD.fasta chp50_PpWT.fastq
% midhr -ptd 1000 -job i14h3r4_1 /DBdir/MT_MOD.fasta chp50_PpWT.fastq

Here the last two arguments are reference sequence and read data as same as ispmap (2.0).
Please note that the job name has the same suffix as ispmap
(_0 for chloroplast and _1 for mitochondorion).
The value specified with the ptd option is the distance threshold to distinguish
a pair of reads facing each other is normal (closer) or abnormal (distant).
There are four output files, .pdist, .pdist2, .pdist3, .pdist4.

pdist:  Mapped positions of pair reads are more than -ptd value appart.
        (Abnormal pair, regardless of their directions)
pdist2: Mapped positions of pair reads are within -ptd value appart,
        and faced to each other. (Normal pair)
pdist3: Mapped positions of pair reads are within -ptd value appart,
        and pointing the same direction. (Abnormal pair)
pdist4: All abnormal pairs, pdist + pdist3.

-------------------------------------------------------------------------------------------

3.1.1 Detection of long (more than 50bp) repeats in the reference

Some of the abnormal pairs in .pdist (.pdist4) file can be artifacts caused by
the presence of long identical sequence pairs in reference genome.
To decrease this kind of artifact, first we prepare the list of identical sequences
longer than threshfold length (50 bp in this example).

% idss  -i 8 -m 50 CH_MOD.fasta > CHi8m50.ids
% idss  -i 8 -m 50 MT_MOD.fasta > CHi8m50.ids

The threshold value is specified with '-m' option, and '-i' option specifies
the index value used for the search.
Index value should be smaller than 15, and also, smaller than the threshold value.
The idss outputs the results into standard output, so it should be redirected
into a file (CHi8m50.ids).

-------------------------------------------------------------------------------------------

3.1.2 Removal of artifacts caused by repeats in reference

Based on the ids file obtained, we can remove some artifact using 'exclude_id_pairs'.

% exclude_id_pairs chp50_PpWT_0.pdist CHi8m50.ids > chp50_PpWT_0.excl.pdist
% exclude_id_pairs chp50_PpWT_1.pdist MTi8m50.ids > chp50_PpWT_1.excl.pdist

Here again, the output is redirected.
The positions of read pairs suggesting the same rearrangement may have similar values,
but usually not exactly identical.
Therefore we need to cluster the read pairs suggesting the same rearrangement
based on the mapped positions of pair reads.

% sort_pdist chp50_PpWT_0.excl.pdist
% sort_pdist chp50_PpWT_1.excl.pdist

'sort_pdist' generates a .pe file (here we get chp50_PpWT_0.excl.pdist.pe and
chp50_PpWT_1.excl.pdist.pe), that contains the list of clusters of the paired reads.

-------------------------------------------------------------------------------------------

3.1.3 Circos-type drawing of links by paired end information

Using this .pe file and .peb file obtained by ispmap (2.0),
we can generate a Circos-like map as follows.

% circmaps -rpb chp50_PpWT.fastq_i14h2r3_0.peb \
           -rgb /DBdir/CH_MOD.gb \
           -rfa /DBdir/CH_MOD.fasta \
           -pe \
           -bls 1.0 \
           -psc 2 \
           -drm 5 \
           chp50_PpWT.fastq_i14h2r3_0.excl.pdist.pe

% circmaps -rpb chp50_PpWT.fastq_i14h2r3_1.peb \
```

```
          -rgb /DBdir/MT_MOD.gb \
          -rfa /DBdir/MT_MOD.fasta \
          -pe \
          -bls 1.0 \
          -psc 2 \
          -drm 5 \
          chp50_PpWT.fastq_i14h2r3_1.excl.pdist.pe
```

These are 2 long single line commands separated by backslash (\).
The argument in the last line is the .pe file just generated(3.1.2).
Following is the description of the options.

```
-rpb ~ :  Specifies the .peb file name created in 2.0
-rgb ~ :  Specifies the gen bank(.gb) file name. Used to plot the gene position
-rfa ~ :  Specifies the reference genome fasta file.
-pe    :  Paired end analysis reading the .pe file
-bls   :  Scale factor for the plot of predicted depth increase/decrease by links.
          (default 1.0)
-psc   :  Scale factor for the depth plot by .peb. (default 1.0)
-drm   :  Draw method. 5 is the only option for .pe, at the moment.
```

This will generate a postscript file with the Circos-like diagram.

3.2 Junction read analysis (midhr, circmaps)

3.2.1 Detection of Junction reads, Formation of Junction read clusters and Rearrangement Prediction

Based on the mapping result of untruncated reads (1.0),
'midhr' detects the junction reads/cluster and rearrangements suggested by them.

```
% midhr -cl 13 -cd 3 -job i14h75r10_0 /DBdir/CH_MOD.fasta PpWT.fastq
```

The detection of junction and clustering junction reads is somewhat complicated.
Please refer to the description in our paper, or contact us for further information.
You can control the sensitivity by 2 criteria.

```
-cl  # : Minimum length of the mismatch consensus sequence
         (the longer the more sensitive)
-cd  # : Minimum number of reads to form mismatch consensus
         (the more the more sensitive)
-job ~ : jobname with suffix (_0 for the first, _1 for the second reference)
```

The last two arguments are the file names of the reference sequence and the
read sequence.

Output files with different suffix contains the different kind of mutation.

```
.del : Simple deletion less than 50 bps.
.ins : Simple insertion less than 50 bps.
.hr  : Homologous rearrangement with more than 2bp microhomology
.hmr : Homologous rearrangement (in both direction).
.pal : Homologous rearrangement caused by (quasi) palindrome less than 70bp.
       (Hairpin turn rearrangement)
.unk : Rearrangement without microhomology
.arr : .hr + .unk + .del + .ins
```

Output files with following suffix contains the information about the status
of junction clusters.

```
.jcr : Junction read information. Shows alignment with reference and mismatch
       start point call.
.jcc : Junction cluster information. Shows alignment of mismatch consensus
       sequences of all junction reads in cluster.
.jcs : List of junction clusters with mismatch starting position and search
       results of the mismatch consensus sequence on reference.
```

3.2.1.1 Rebuilding .hmr file

The .hmr file generated in the previous section 3.2.1 include the junction read that may include
several mismatches. To improve the reliability of .hmr output, we can use program h2h to generate
.hmr file from .hr file which is more reliable. With the .hr file PpWT.fastq_i14h75r10_0.hr, type

```
% h2h PpWT.fastq_i14h75r10_0.hr
```

This will generate a .hmr file PpWT.fastq_i14h75r10_0.hr.hmr
Sorting the second column of this file in numerical order will provide an outpu which is easier to
recognize.

```
% sort -k 2 PpWT.fastq_i14h75r10_0.hr.hmr > PpWT_CH.hmr
```

3.2.2 Drawing Circos-like map by junction read analysis

Using circmaps we can generate Circos-like diagram as similar fashion as

paired-end analysis (3.1.3).
This time run it without -pe option, and specify 6 for -drm option,
and put .hr, .arr, or .unk file as the last argument.

```
circmaps -rpb chp50_PpWT.fastq_i14h2r3_0.peb \
           -rgb /DBdir/CH_MOD.gb \
           -rfa /DBdir/CH_MOD.fasta \
           -bls 1.0 \
           -psc 2 \
           -drm 6 \
           PpWT.fastq_i14h75r10_0.arr
```

This will generate a postscript file of the diagram. PpWT.fastq_i14h75r10_0.arr.ps

----
----

4. Comparison of rearrangements/mutations (links) among experiments
   (comp_del, comp_ins, comp_hr, comp_hmr, comp_pal, comp_unk, comp_pe)

Some of the rearrangements are shared among experiments,
and some are found only in some experiments.
In order to compare .del, .ins, .hr, .hmr, .pal, .unk, .pe files among experiments
and organize the identical rearrangements/mutations, we prepared small programs:comp_del,
comp_ins, comp_hr, comp_hmr, comp_pal, comp_unk, comp_pe, respectively.

Their usages are similar, and here we show how to use comp_del.
First we need to prepare a list of .del files as follows.

```
========================== del.list =============================
PpWT.fq_i11h35r36immap_0.del   40223458
Mut1.fq_i11h35r36immap_0.del   37033756
Mut2.fq_i11h35r36immap_0.del   42008745
=================================================================
```

The list consists of two column. The left column is the .del file name
and the right column is the integer number
to calibrate the intensity among experiments (such as the number of total/mapped reads).
The first line is the standard and typically is the wild type experiment.

```
% comp_del del.list
```

Program ’comp_del’ takes the prepared list file name (del.list) as the only argument.
It generates two outputs with suffixes ’.out’ and ’.out2’.
As for .ins, .del, .pal, .hmr, the output files are cvs (comma separated),
and for .hr, .unk, .pe, the output files are fixed length space separated files.
Columns in right hand side are the number of junction reads indicating
the rearrangement/mutaion (.out) for each experiment,
and the numbers calibrated by the second column of the list file (.out2).
For instance, the example del.list file contains three lines, PpWT, Mut1 and Mut2,
the 3 column from the right corresponds to the PpWT, Mut1, and Mut2, respectively.

Definitions of columns on the left side are as follows.

```
del : 1. Genome position, 2. Deletion length
ins : 1. Genome position, 2. Insertion length, 3. Inserted sequence
pal : 1. Genome position(left) 2. Genome position (right)
      3. (quasi) Palindrome sequence
hmr : 1. Genome position(homologous sequence 1 left)
      2. Genome position (homologous sequence 1 right)
      3. Genome position(homologous sequence 2 left)
      4. Genome position (homologous sequence 2 right)
      5. Homologous sequence

unk : 1. Genome position (rearrange from)
      2. Genome position (rearrange to)
hr  : 1. Genome position (rearrange from)
      2. Genome position (rearrange to)
      3. Homologous sequence
pe  : 1. Genome position (rearrange from)
      2. Genome position (rearrange to)
```

(For pe, the positions are the mapped positions of the seed pair of the cluster,
indicating the same rearrangement.)

----

5. Depth plot (calibrated in comparison with WT experiment)
   (pop_comp)

Mapping depth plot of each experiment was obtained with ’ispmap’ (2.1).
’ispmap’ also creates .peb file that contains the number of read mapped
at each position of reference sequence as a text file.
Here we describe the procedure to create a plot calibrated by standare
(wild type) experiment.
In our case, we used the number of reads mapped on nuclear chromosome
for each experiment to calculate the scale factor.
We assumed, gene knowckout mutant may have decreased/increased read count
for each organella, while the number of reads mapped on nuclear chromosome
should stay constant. Therefore we divide the number of reads mapped
on nuclear chromosome of wild type, by the number of reads mapped on nuclear
chromosome of knock out mutant, to obtain the scale factor (0.588 in our example).

```
% pop_comp -sf 0.588 WT.fastq_i14h2r3_0.peb MUT1.fastq_i14h2r3_0.peb
% pop_comp -sf 0.588 WT.fastq_i14h2r3_1.peb MUT1.fastq_i14h2r3_1.peb
```

The value after '-sf' option is the scale factor, and the last two arguments are .peb files of wild type and mutant.
This will create a postscript file 'MUT1.fastq_i14h2r3_0.peb.ps' that contains, depth plot of wild type, mutant, and the normalized plot.

----

6. Histogram of number of repeats longer/shorter than a threshold
   (reptile)

Count the number of repeats longer than -min value and shorter than -max value and draw a histgram along the genome sequence.

```
% reptile -ph -ps -idx 10 -min 11 -max 100 MT.fasta > MT_11_100.out
```

The program generates output file with suffix .pos, .dst, .hmp.
Here we use MT.fasta.pos to draw a histgram using R.

```
Launch R and load science library.
> library(MASS)
Load .pos file.
> MT_11_100 = scan("/Users/.../MT.fasta.pos")
Specify output ps file name.
> postscript("MT_11_100.ps")
Draw histogram
> truehist(MT_11_100,prob=FSLSE,nbins = 50)
Create .ps file
> dev.off()
```

'MT.fasta.ps' is generated.

----

7. Plot histogram of rearrangement position along genome, based on the midhr output (.hr)
   (hr_count)

Based on the junction analysis of 'midhr' (.hr output), 'hr_count' creates a histgram of the number of homologous rearrangement along the genome sequence.

```
% hr_count Mut1.fastq_i14h75r10_0.hr
```

This will generate three files Mut1.fastq_i14h75r10_0.hr.3_5.pos,
Mut1.fastq_i14h75r10_0.hr.6_10.pos and Mut1.fastq_i14h75r10_0.hr.11_100.pos
The output file Mut1.fastq_i14h75r10_0.hr.3_5.pos contains the positions of rearrangement with which the length of homologous sequnce is from 3 to 5 bps.
The other output files contains positions of rearrangements with the length of homologous sequences, from 6 to 10, and 11 to 100.

To draw the histogram from one of these outputs 'Mut1.fastq_i14h75r10_0.hr.3_5.pos'

```
Launch R and load science library.
> library(MASS)
Load .pos file.
> Mut1_3_5 = scan("/Users/.../Mut1.fastq_i14h75r10_0.hr.3_5.pos")
Specify output ps file name.
> postscript("Mut1_3_5.ps")
Draw histogram
> truehist(Mut1_3_5,prob=FALSE,nbins = 50)
Create .ps file
> dev.off()
```

'Mut1_3_5.ps' is generated.

