# Supplementary material for: Ultra-deep sequencing reveals dramatic alteration of organellar genomes in Physcomitrella patens due to biased asymmetric recombination
Source: Commun Biol. 2021 May 27;4:633. doi: 10.1038/s42003-021-02141-x (PMC8159992; doi:10.1038/s42003-021-02141-x)
Supplement: Supplementary file 3 — Description of Additional Supplementary Files [file 42003_2021_2141_MOESM3_ESM.pdf]

Description of Additional Supplementary Files

File name: Supplementary data 1
Description: Ratio of 150 bp or 100 bp reads with allowing mismatches.

File name: Supplementary data 2
Description: List of repeats identified in chloroplast and mitochondrial DNA.

File name: Supplementary Data 3
Description: Lists of junction read clusters and paired-end read clusters.

File name: Supplementary Data 4
Description: Source dataset for Figure 1.
